# Time–outcome relationship in acute large-vessel occlusion exists across all ages: subanalysis of RESCUE-Japan Registry 2

**DOI:** 10.1038/s41598-021-92100-7

**Published:** 2021-06-17

**Authors:** Kenichi Todo, Shinichi Yoshimura, Kazutaka Uchida, Hiroshi Yamagami, Nobuyuki Sakai, Haruhiko Kishima, Hideki Mochizuki, Masayuki Ezura, Yasushi Okada, Kazuo Kitagawa, Kazumi Kimura, Makoto Sasaki, Norio Tanahashi, Kazunori Toyoda, Eisuke Furui, Yuji Matsumaru, Kazuo Minematsu, Takaya Kitano, Shuhei Okazaki, Tsutomu Sasaki, Manabu Sakaguchi, Masatoshi Takagaki, Takeo Nishida, Hajime Nakamura, Takeshi Morimoto, RESCUE-Japan Registry 2 Investigators, Kazunori Toyoda, Masataka Takeuchi, Masafumi Morimoto, Toshiyuki Onda, Masunari Shibata, Shinichi Yoshimura, Nobuyuki Sakai, Takahiro Ohta, Keisuke Imai, Ryo Itabashi, Masayuki Ezura, Taro Yamashita, Norihito Fukawa, Naoto Kimura, Ryosuke Doijiri, Hajime Ohta, Yukiko Enomoto, Chisaku Kanbayashi, Ikuya Yamaura, Hideyuki Ishihara, Yuki Kamiya, Makoto Hayase, Kouhei Nii, Junya Kobayashi, Hiroaki Yasuda, Ryushi Kondo, Daisuke Yamamoto, Manabu Sakaguchi, Junichiro Satomi, Yoshiki Yagita, Akira Handa, Atsushi Shindo, Nagayasu Hiyama, Naoki Toma, Tomoyuki Tsumoto, Kazumi Kimura, Wataro Tsuruta, Keigo Matsumoto, Yoshihiro Kiura, Takaaki Yamazaki, Taketo Hatano, Yoshihisa Matsumoto, Takao Kojima, Norio Ikeda, Shigeyuki Sakamoto, Hiroyuki Ohnishi, Koichi Haraguchi, Naoyuki Uchiyama

**Affiliations:** 1 Stroke Center, Osaka University Hospital, Suita, Osaka Japan; 2 Department of Neurosurgery, Hyogo College of Medicine, Nishinomiya, Hyogo Japan; 3 Department of Stroke Neurology, National Hospital Organization Osaka National Hospital, Osaka, Japan; 4 Department of Cerebrovascular Medicine, National Cerebral and Cardiovascular Center, Suita, Osaka Japan; 5 Department of Neurosurgery, Kobe City Medical Center General Hospital, Kobe, Hyogo Japan; 6 Department of Neurosurgery, National Hospital Organization Sendai Medical Center, Sendai, Miyagi Japan; 7 Cerebrovascular Center, National Hospital Organization Kyushu Medical Center, Fukuoka, Japan; 8 Department of Neurology, Tokyo Women’s Medical University, Tokyo, Japan; 9 Department of Neurological Science, Graduate School of Medicine, Nippon Medical School, Tokyo, Japan; 10 Institute for Biomedical Sciences, Iwate Medical University, Morioka, Iwate Japan; 11 Department of Neurology, Saitama Medical University International Medical Center, Hidaka, Saitama Japan; 12 Department of Stroke Neurology, Saiseikai Toyama Hospital, Toyama, Japan; 13 Division for Stroke Prevention and Treatment, Department of Neurosurgery, University of Tsukuba, Tsukuba, Ibaraki Japan; 14 Department of Clinical Epidemiology, Hyogo College of Medicine, Nishinomiya, Hyogo Japan; 15 Department of Cerebrovascular Medicine, National Cerebral and Cardiovascular Center, Suita, Osaka Japan; 16 Department of Neurosurgery, Seisho Hospital, Odawara, Kanagawa Japan; 17 Department of Neurosurgery, Yokohama Shintoshi Neurosurgical Hospital, Yokohama, Kanagawa Japan; 18 Department of Neurosurgery, Sapporo Shiroishi Memorial Hospital, Sapporo, Hokkaido Japan; 19 Department of Neuroendovascular Treatment, Japanese Red Cross Ise Hospital, Ise, Mie Japan; 20 Department of Neurosurgery, Hyogo College of Medicine, Nishinomiya, Hyogo Japan; 21 Department of Neurosurgery, Kobe City Medical Center General Hospital, Kobe, Hyogo Japan; 22 Department of Neurosurgery, Tokyo Metropolitan Tama Medical Center, Fuchu, Tokyo Japan; 23 Department of Neuroendovascular Treatment, Japanese Red Cross Kyoto Daiichi Hospital, Kyoto, Japan; 24 Department of Stroke Neurology, Kohnan Hospital, Sendai, Miyagi Japan; 25 Department of Neurosurgery, National Hospital Organization Sendai Medical Center, Sendai, Miyagi Japan; 26 Department of Neurosurgery, Shimizu Hospital, Kyoto, Japan; 27 Department of Neurosurgery, Kinki University, Osaka-Sayama, Osaka Japan; 28 Department of Neurosurgery, Iwate Prefectural Central Hospital, Morioka, Iwate Japan; 29 Department of Neurology, Iwate Prefectural Central Hospital, Morioka, Iwate Japan; 30 Department of Neurosurgery, Miyakonojo Medical Association Hospital, Miyakonojo, Miyazaki Japan; 31 Department of Neurosurgery, Gifu University Hospital, Gifu, Japan; 32 Department of Neurosurgery, Kawasaki Saiwai Hospital, Kawasaki, Kanagawa Japan; 33 Department of Neurosurgery, Yoshida Hospital and Cerebrovascular Research Institute, Kobe, Hyogo Japan; 34 Department of Neurosurgery, Yamaguchi University Hospital, Ube, Japan; 35 Department of Neurology, Showa University Koto-Toyosu Hospital, Tokyo, Japan; 36 Department of Neurosurgery, Fukui Red Cross Hospital, Fukui, Japan; 37 Department of Neurosurgery, Fukuoka University Chikushi Hospital, Chikushino, Fukuoka Japan; 38 Department of Vascular Neurology, Osaka-Minami Medical Center, Kawachinagano, Osaka Japan; 39 Department of Neurosurgery, Yamaguchi Prefectural Grand Medical Center, Yamaguchi, Japan; 40 Department of Neurosurgery, Kitasato University Hospital, Sagamihara, Kanagawa Japan; 41 Department of Neurology, Osaka University Hospital, Suita, Osaka Japan; 42 Department of Neurosurgery, Tokushima University Hospital, Tokushima, Japan; 43 Department of Stroke Medicine, Kawasaki Medical School Hospital, Kurashiki, Okayama Japan; 44 Department of Neurosurgery, Kurashiki Central Hospital, Kurashiki, Okayama Japan; 45 Department of Neurological Surgery, Kagawa University Faculty of Medicine, Miki, Kagawa Japan; 46 Department of Neurosurgery, Goshi Hospital, Amagasaki, Hyogo Japan; 47 Department of Neurosurgery, Mie University Hospital, Tsu, Mie Japan; 48 Cerebrovascular Center, National Hospital Organization Kyushu Medical Center, Fukuoka, Japan; 49 Department of Neurological Science, Graduate School of Medicine, Nippon Medical School, Tokyo, Japan; 50 Department of Neurology, Toranomon Hospital, Tokyo, Japan; 51 Department of Neurosurgery, Kobe Central Hospital, Kobe, Hyogo Japan; 52 Department of Neurology, Hiroshima Prefectural Hospital, Hiroshima, Japan; 53 Department of Neurosurgery, Hakodate Neurosurgical Hospital, Hakodate, Hokkaido Japan; 54 Department of Neurosurgery, Kokura Kinen Hospital, Kitakyūshū, Fukuoka Japan; 55 Department of Neurosurgery, Tanushimaru Central Hospital, Kurume, Fukuoka Japan; 56 Department of Neurosurgery, Japanese Red Cross Nagoya Daini Hospital, Nagoya, Aichi Japan; 57 Department of Neurosurgery, Ube-Kohsan Central Hospital, Ube, Yamaguchi Japan; 58 Department of Neurosurgery, Hiroshima University Hospital, Hiroshima, Japan; 59 Department of Neurosurgery, Osaka Medical College Hospital, Takatsuki, Osaka Japan; 60 Department of Neurosurgery, Hakodate Shintoshi Hospital, Hakodate, Hokkaido Japan; 61 Department of Neurosurgery, Kanazawa University, Kanazawa, Japan

**Keywords:** Neurology, Neurological disorders, Stroke

## Abstract

Early reperfusion after endovascular thrombectomy is associated with an improved outcome in ischemic stroke patients; however, the time dependency in elderly patients remains unclear. We investigated the time–outcome relationships in different age subgroups. Of 2420 patients enrolled in the RESCUE-Japan Registry 2 study, a study based on a prospective registry of stroke patients with acute cerebral large-vessel occlusion at 46 centers, we analyzed the data of 1010 patients with successful reperfusion after endovascular therapy (mTICI of 2b or 3). In 3 age subgroups (< 70, 70 to < 80, and ≥ 80 years), the mRS scores at 90 days were analyzed according to 4 categories of onset-to-reperfusion time (< 180, 180 to < 240, 240 to < 300, and ≥ 300 min). In each age subgroup, the distributions of mRS scores were better with shorter onset-to-reperfusion times. The adjusted common odds ratios for better outcomes per 1-category delay in onset-to-reperfusion time were 0.66 (95% CI 0.55–0.80) in ages < 70 years, 0.66 (95% CI 0.56–0.79) in ages 70 to < 80 years, and 0.83 (95% CI 0.70–0.98) in ages ≥ 80 years. Early reperfusion was associated with better outcomes across all age subgroups. Achieving early successful reperfusion is important even in elderly patients.

## Introduction

Randomized clinical trials have demonstrated that endovascular thrombectomy for acute large-vessel occlusion has beneficial effects on 90-day outcomes^1–6^. Furthermore, a shorter time to successful reperfusion after endovascular thrombectomy has been demonstrated to be associated with better functional outcomes^7–10^. Although older age is a strong predictor of worse outcomes in stroke patients^11^, the treatment effect of endovascular thrombectomy has been proven to be consistent across all ages^1^; thus, guidelines recommend endovascular therapy even in octogenarians^12^. However, the effect of a shorter time to reperfusion in elderly patients remains controversial^13,14^. Therefore, we sought to identify the time–outcome relationship in elderly patients. For this purpose, we analyzed the association of onset-to-reperfusion time (ORT) with outcomes in different age subgroups using a large practice-based database.

## Methods

### Ethics statement

This study complied with the Declaration of Helsinki guidelines for investigations involving humans, and all methods were performed in accordance with relevant guidelines and regulations for observational studies. The study design and protocols were approved by the institutional review boards of all participating centers. Approving institutional review boards waived the need for informed consent because we used clinical information obtained in routine clinical practice. The institutional review boards of all participating centers approved the exemption in accordance with the Ethical Guidelines for Medical and Health Research Involving Human Subjects in Japan. The full names of all institutional review boards are as follows: Institutional review boards of Red Cross Ise Hospital, Ube Industries Central Hospital, Ogaki Tokushukai Hospital, Osaka Medical College, Osaka University Hospital, Kagawa University, Kawasaki Medical School Hospital, Kanazawa Medical University Hospital, Kitasato University, Gifu University, Kyushu Medical Center, Red Cross Kyoto Daiichi Hospital, Kinki University, Kurashiki Central Hospital, Kurume University Hospital, Kannan Hospital, Kobe City Medical Center General Hospital, Kokura Memorial Hospital, National Cerebral and Cardiovascular Center, Saiseikai Toyama Hospital, Saiseikai Nagasaki Hospital, Saitama Medical University International Medical Center, Sapporo Medical University, Shimizu Hospital, Juntendo University Hospital, Seisho Hospital, National Hospital Organization Sendai Medical Center, Koseikai Takeda Hospital, Tanushimaru Central Hospital, Tama Medical Center, Tokushima University, Toranomon Hospital, Nagoya University, Red Cross Nagoya Daini Hospital, Nippon Medical School, Hakodate Shintoshi Hospital, Hakodate Neurosurgical Hospital, Hyogo College of Medicine, Hyogo Brain and Heart Center, Hirosaki University, Hiroshima University, Red Cross Fukui Hospital, Fukuoka University Chikushi Hospital, Mazda Hospital, Mie University Hospital, Miyakonojo Medical Association Hospital, Yamaguchi Prefectural Grand Medical Center, Yamaguchi University and Yokohamashintoshi Neurosurgical Hospital.

### Subjects

This study is a post-hoc analysis of RESCUE-Japan Registry 2^15^, which was a prospective multicenter registry that enrolled 2420 patients with acute cerebral large-vessel occlusion at 46 centers in Japan between October 1, 2014 and September 30, 2016. RESCUE-Japan Registry 2 was designed to clarify the generalizability of the effectiveness of endovascular therapy in real-world patients. We enrolled consecutive patients aged ≥ 20 years who were hospitalized within 24 h of the onset of acute cerebral large-vessel occlusion.

### Imaging and endovascular therapy

The diagnostic and treatment modalities were not unified in the RESCUE-Japan Registry 2. The Alberta Stroke Program Early Computerized Tomography Score (ASPECTS) was derived from computed tomography (CT) or magnetic resonance diffusion-weighted imaging (DWI)^16,17^. In patients with stroke in the posterior circulation, we measured the posterior circulation ASPECTS (pc-ASPECTS) using DWI^18^. The treatment modalities were determined by the attending physician. In this study, endovascular therapy included thrombectomy using stent retrievers and/or aspiration catheters, balloon angioplasty, stenting, intra-arterial fibrinolysis, piercing using guidewires and/or microcatheters, or a combination of these treatments, all of which have been approved in Japan. The stent retrievers used in this study were the Solitaire^™^ 2 revascularization device (Covidien, Irvine, CA), the Trevo^®^ ProVue retriever/Trevo^®^ XP ProVue retriever (Stryker, Fremont, CA), and the Revive^®^ retriever (Codman, Raynham, MA). The aspiration catheter used was the Penumbra^®^ system (Penumbra, Alameda, CA). Other devices for endovascular therapy procedures such as stenting or angioplasty were selected by the physicians in charge.

### Variables and measurements

We obtained clinical information of patients from hospital charts. Follow-up information up to 90 days was collected primarily through a review of the hospital charts or by contacting the patients, relatives, and/or physicians. We used the following clinical data for the analyses in the current study: age, sex, vascular risk factors (i.e., atrial fibrillation, hypertension, and diabetes mellitus), pre-stroke modified Rankin Scale (mRS) score^19^, National Institute of Health Stroke Scale (NIHSS) score^20^, blood glucose level on admission, systolic blood pressure on admission, location of occlusion, use of intravenous recombinant tissue plasminogen activator (IV-rtPA), ASPECTS, onset-to-puncture time (OPT), ORT, modified thrombolysis in cerebral infarction (mTICI) score (0, no perfusion; 1, minimal perfusion; 2a, reperfusion of less than half of the previously occluded territory; 2b, reperfusion of more than half of the previously occluded territory; 3, complete reperfusion)^21^, symptomatic intracranial hemorrhage (sICH), and mRS score at 90 days after stroke. sICH was defined as intracranial hemorrhage within 72 h after stroke with neurological worsening of ≥ 4 points in the NIHSS score^22^. OPT and ORT were defined as the duration from the time that the patient was last seen to be well to the groin puncture and the end of the endovascular therapy, respectively. We defined successful reperfusion as an mTICI score of ≥ 2b.

We divided the patients into 3 age subgroups: < 70, 70 to < 80, and ≥ 80 years. Since the time–outcome relationship was demonstrated in patients with OPT of 6 h or less^7–10^, but not in patients with OPT of more than 6 h^23,24^, we focused on the early time window and divided ORT into 4 categories with thresholds of 180, 240, and 300 min. We compared the clinical background characteristics and outcome measurements according to the ORT categories in each age subgroup.

The primary outcome was set as the ordinal score on the mRS score at 90 days after stroke. The secondary outcomes were set as a good outcome (defined as mRS score ≤ 2) and mortality at 90 days after stroke. The safety outcome was defined as the occurrence of an sICH within 72 h after stroke.

### Statistical analyses

We analyzed the database of patients with successful reperfusion after endovascular therapy (mTICI score 2b or 3). We analyzed continuous variables using the Mann–Whitney U test or Kruskal–Wallis test, and expressed them as median values and interquartile ranges. We analyzed categorical data using the chi-square test, and expressed them as numbers and percentages. In each age subgroup, the trends in the distributions of mRS scores were analyzed according to the ORT categories using the Jonckheere–Terpstra test. We developed multivariate logistic regression models to assess the association between ORT and outcomes by adjusting for the following clinically relevant factors: ASPECTS (≥ 6 or < 6), NIHSS score, pre-stroke mRS score, use of IV-rtPA, site of the main occlusions (anterior or posterior circulation), and vessels of the main occlusions (internal carotid artery [ICA] and M1 segment of the middle cerebral artery occlusion or other arteries). ORT was entered as a categorical or a continuous variable. Because the threshold of 6 for both anterior circulation ASPECTS and pc-ASPECTS was reported to show good discrimination ability^25,26^, we dichotomized both scores according to whether they were above or below 6. If both CT and DWI were performed before endovascular therapy, we used ASPECTS on DWI in patients with anterior circulation occlusion. We used pc-ASPECTS in patients with stroke in the posterior circulation. To assess potential selection bias, we performed sensitivity analyses. First, we developed multivariate logistic regression models using the multiple imputation method (Supplementary Methods). Second, we analyzed the database of patients with an OPT of ≤ 6 h. Third, we developed multivariate logistic regression model using ASPECTS on CT in patients with both ASPECTS on DWI and CT.

Statistical significance was set at *P* < 0.05. We conducted all analyses with R software using the rms package (version 3.3.3; F Foundation for Statistical Computing, Vienna, Austria)^27^.

## Results

### Baseline characteristics

**Figure 1:**
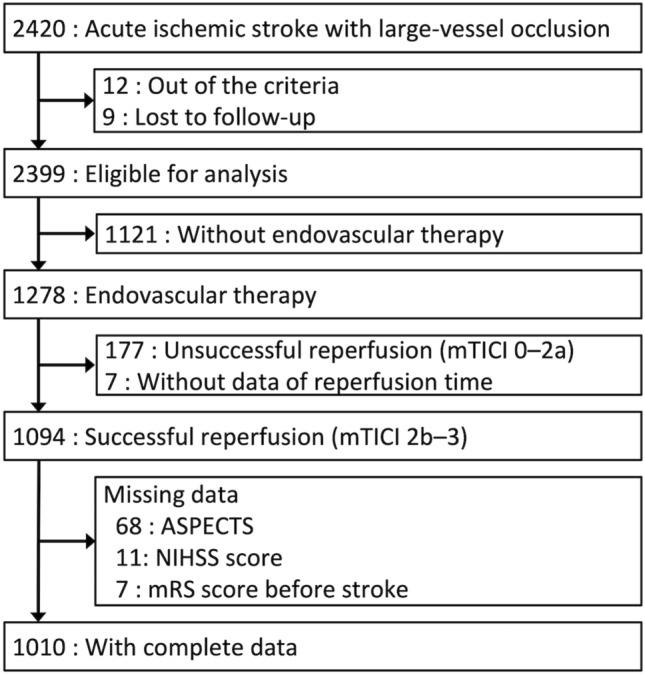
Flowchart of the study population. *ASPECTS* Alberta Stroke Program Early Computed Tomography Score, *mTICI* modified thrombolysis in cerebral infarction, *NIHSS* National Institutes of Health Stroke Scale.

Among 2420 enrolled patients, 21 patients were excluded because 12 patients did not meet the eligibility criteria and 9 patients were lost to follow-up. Of the remaining 2399 patients, 1278 patients underwent endovascular therapy. Among them, 177 patients with unsuccessful reperfusion (mTICI 0–2a) and 7 patients without reperfusion time data were excluded. Patients without data of ASPECTS, NIHSS score, or mRS score before stroke were also excluded. Thus, 1010 patients were included in the analysis (Fig. 1).

Clinical characteristics according to the age subgroups are shown in Table 1. The proportions of patients with female sex, hypertension, atrial fibrillation, and pre-stroke mRS score > 2 were higher in the elderly subgroups. The NIHSS score and systolic blood pressure were higher in elderly patients. The proportion of patients with diabetes mellitus, IV-rtPA use, and ASPECTS ≥ 6 were not different among the age subgroups. The blood glucose level, location of the occlusion, OPT, and ORT were not different among the age subgroups.

**Table 1:** Clinical characteristics according to age.

	Total (N = 1010)	Age (years)			*P* value
		< 70 (N = 303)	≥ 70 to < 80 (N = 337)	≥ 80 (N = 370)	
Age, median (IQR), y	76 (68–82)	64 (55–67)	75 (73–77)	85 (82–87)	< 0.0001
Male sex, No. (%)	603 (60%)	226 (75%)	219 (65%)	158 (43%)	< 0.0001
Hypertension, No. (%)	584 (58%)	140 (46%)	204 (61%)	240 (64%)	< 0.0001
Diabetes mellitus, No. (%)	193 (19%)	63 (21%)	71 (21%)	69 (16%)	0.15
Atrial fibrillation, No. (%)	533 (53%)	119 (39%)	182 (54%)	232 (63%)	< 0.0001
Pre-stroke mRS score ≥ 2, No. (%)	897 (89%)	291 (96%)	310 (92%)	296 (80%)	< 0.0001
NIHSS score, median (IQR)	18 (13–23)	16 (12–22)	18 (13–23)	18 (14–23)	0.0021
Blood glucose, median (IQR), mg/mL (N = 984)	128 (110–156)	126 (111–156)	129 (110–156)	127 (110–154)	0.98
Systolic blood pressure, median (IQR), mmHg (N = 981)	154 (135–170)	150 (128–167)	152 (136–170)	160 (140–173)	< 0.0001
**Location of occlusion**					
ICA, No. (%)	346 (34%)	89 (29%)	123 (36%)	134 (36%)	0.101
M1 segment MCA, No. (%)	424 (42%)	129 (43%)	140 (42%)	155 (42%)	0.97
M2 segment MCA, No. (%)	177 (18%)	62 (20%)	50 (15%)	65 (18%)	0.17
M3 segment MCA, No. (%)	6 (1%)	3 (1%)	2 (1%)	1 (0%)	0.48
A1 segment ACA, No. (%)	3 (0%)	0 (0%)	2 (1%)	1 (0%)	0.38
A2 segment ACA, No. (%)	12 (1%)	3 (1%)	4 (1%)	5 (1%)	0.91
BA, No. (%)	177 (8%)	27 (9%)	29 (9%)	21 (6%)	0.21
VA, No. (%)	8 (1%)	6 (2%)	1 (0%)	1 (0%)	0.020
P1 segment PCA, No. (%)	5 (0%)	3 (1%)	0 (0%)	1 (0%)	0.044
P2 segment PCA, No. (%)	2 (0%)	0 (0%)	1 (0%)	1 (0%)	0.65
IV-rtPA therapy, No. (%)	482 (48%)	158 (52%)	163 (48%)	161 (44%)	0.080
ASPECTS on CT, median (IQR) (N = 638)	10 (7–10)	10 (8–10)	10 (8–10)	10 (8–10)	0.82
ASPECTS on DWI, median (IQR) (N = 788)	7 (6–8)	7 (6–8)	8 (6–9)	7 (6–9)	0.12
pc-ASPECTS, median (IQR) (N = 80)	7 (6–8)	7 (6–8)	7 (6–8)	7 (6–8)	0.98
ASPECTS^a^ ≥ 6, No. (%)	843 (83%)	250 (83%)	27 (82%)	317 (86%)	0.35
Onset-to-puncture time, median (IQR), minutes	205 (135–355)	205 (135–320)	200 (135–345)	215 (140–400)	0.11
Onset-to-reperfusion time, median (IQR), minutes	270 (195–435)	265 (185–385)	260 (185–410)	280 (205–475)	0.060

*ACA* anterior cerebral artery, *ASPECTS* Alberta Stroke Program Early Computed Tomography Score, *BA* basilar artery, *CT* computed tomography, *DWI* diffusion-weighted imaging, *ICA* internal carotid artery, *IV-rtPA* intravenous recombinant tissue plasminogen activator, *IQR* interquartile range, *MCA* middle cerebral artery, *mRS* modified Rankin Scale, *NIHSS* National Institutes of Health Stroke Scale, *PCA* posterior cerebral artery, *VA* vertebral artery.

^a^ASPECTS was derived from CT or DWI. If both CT and DWI were performed before endovascular therapy, ASPECTS on DWI was used for the analysis. In patients with stroke in the posterior circulation, posterior circulation ASPECTS on DWI was used for the analysis.

The clinical characteristics according to the ORT categories in each age subgroup are shown in Supplementary Tables S1–S3. The proportion of patients with atrial fibrillation and an NIHSS score were higher in the shorter ORT categories in patients aged ≤ 80 years. The proportion of patients with IV-rtPA use was lower in those with an ORT > 300 min. The proportion of patients with ASPECTS ≥ 6 in patients aged < 70 years and the proportion of patients with ICA or M1 segment occlusion in patients aged 70 to < 80 years was different among the ORT categories.

### Primary outcomes

**Figure 2:**
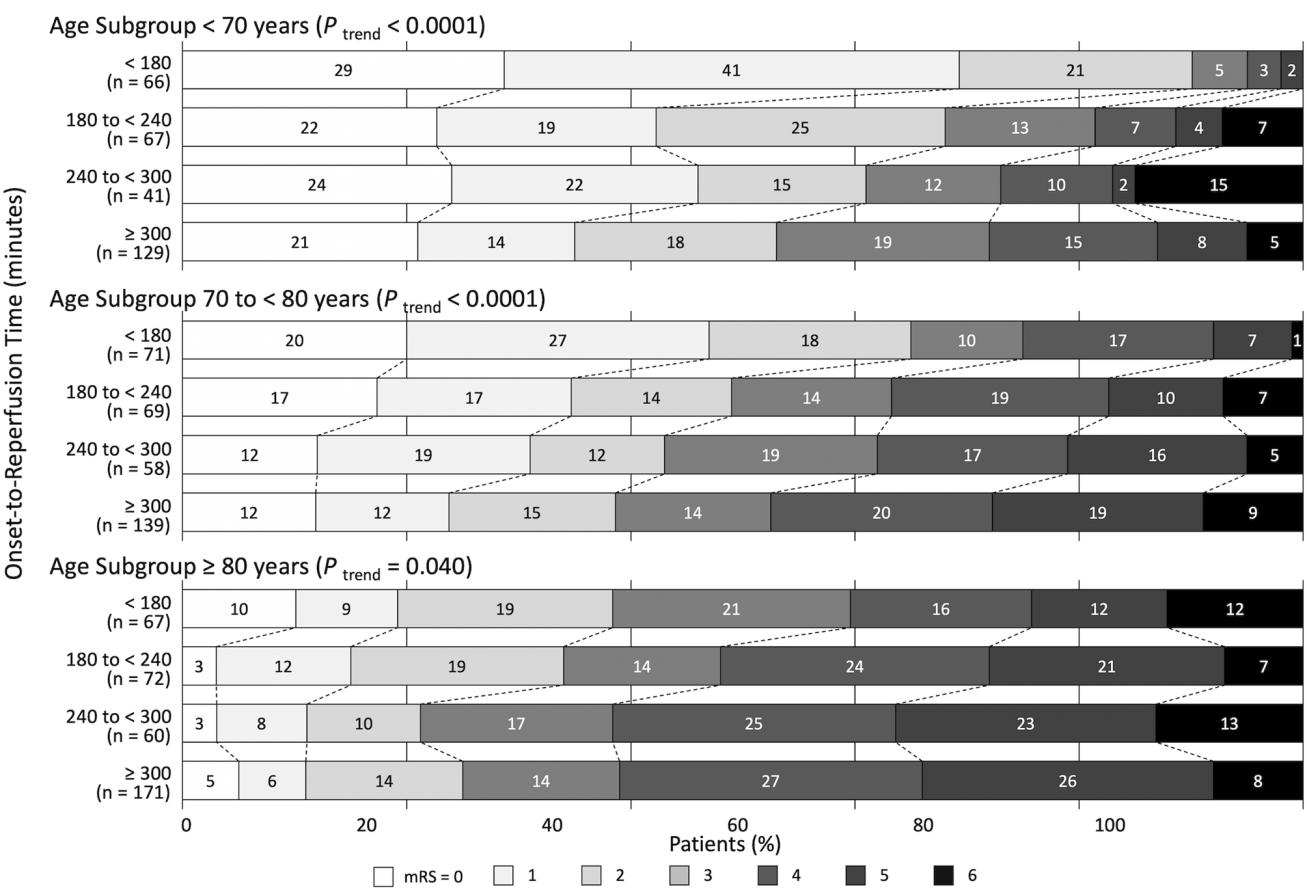
Distributions of the modified Rankin Scale scores at 90 days according to onset-to-reperfusion time categories in each age subgroup. In each age subgroup, onset-to-reperfusion time was associated with better outcomes.

The shorter ORT categories were associated with better distributions of mRS scores at 90 days in each age subgroup (Fig. 2). The adjusted common odds ratios for better outcomes per 1-category delay in ORT were 0.66 (95% confidence interval [CI], 0.55–0.80) in patients aged < 70 years, 0.66 (95% CI 0.56–0.79) in patients aged 70 to < 80 years, and 0.83 (95% CI 0.70–0.98) in patients aged ≥ 80 years (Table 2). These adjusted common odds ratios were significantly different among age subgroups (interaction *P* = 0.031).

**Table 2:** Adjusted odds ratios for outcomes according to ORT.

Outcome	Subjects	Effect variable	Age subgroups	Adjusted values^a^ (95% CI)	*P* for interaction
**Primary outcome**					
mRS score at 90 days	1010 patients with mTICI scores ≥ 2b	Common odds ratios per 1-category delay in ORT^b^	< 70 years (N = 303)	0.66 (0.55–0.80)	
			70 to < 80 years (N = 337)	0.66 (0.56–0.79)	0.031
			≥ 80 years (N = 370)	0.83 (0.70–0.98)	
**Secondary outcomes**					
mRS score ≤ 2 at 90 days	897 patients with mTICI scores ≥ 2b and pre-stroke mRS score ≤ 2	Odds ratios per 1-category delay in ORT^b^	< 70 years (N = 291)	0.52 (0.39–0.68)	
			70 to < 80 years (N = 310)	0.62 (0.49–0.78)	0.016
			≥ 80 years (N = 296)	0.76 (0.60–0.96)	
Mortality at 90 days	1010 patients with mTICI scores ≥ 2b	Odds ratios per 1-category delay in ORT^b^	< 70 years (N = 303)	1.36 (0.82–2.26)	
			70 to < 80 years (N = 337)	1.59 (1.01–2.50)	0.054
			≥ 80 years (N = 370)	0.88 (0.63–1.22)	
**Safety outcome**					
sICH within 72 h	1010 patients with mTICI scores ≥ 2b	Odds ratios per 1-category delay in ORT^b^	< 70 years (N = 303)	0.98 (0.57–1.69)	
			70 to < 80 years (N = 337)	1.21 (0.72–2.03)	0.75
			≥ 80 years (N = 370)	0.90 (0.58–1.41)	

*CI* confidence interval, *mRS* modified Rankin Scale, *mTICI* modified thrombolysis in cerebral infarction, *ORT* onset-to-reperfusion time, *sICH* symptomatic intracranial hemorrhage.

^a^Adjustment was made for the Alberta Stroke Program Early Computed Tomography Score (ASPECTS) (≥ 6 or < 6), National Institutes of Health Stroke Scale score, pre-stroke modified Rankin Scale score, use of intravenous recombinant tissue plasminogen activator, site of the main occlusions (anterior or posterior circulation), and vessels of the main occlusions (internal carotid artery and M1 segment of the middle cerebral artery occlusion or other arteries). ASPECTS was derived from computed tomography (CT) or magnetic resonance diffusion-weighted imaging (DWI). If both CT and DWI were performed before endovascular therapy, ASPECTS on DWI was used for the analysis. In patients with stroke in the posterior circulation, posterior circulation ASPECTS on DWI was used for the analysis.

^b^ORT was divided into four categories: < 180, 180 to < 240, 240 to < 300, and ≥ 300 min.

### Secondary outcomes

**Figure 3:**
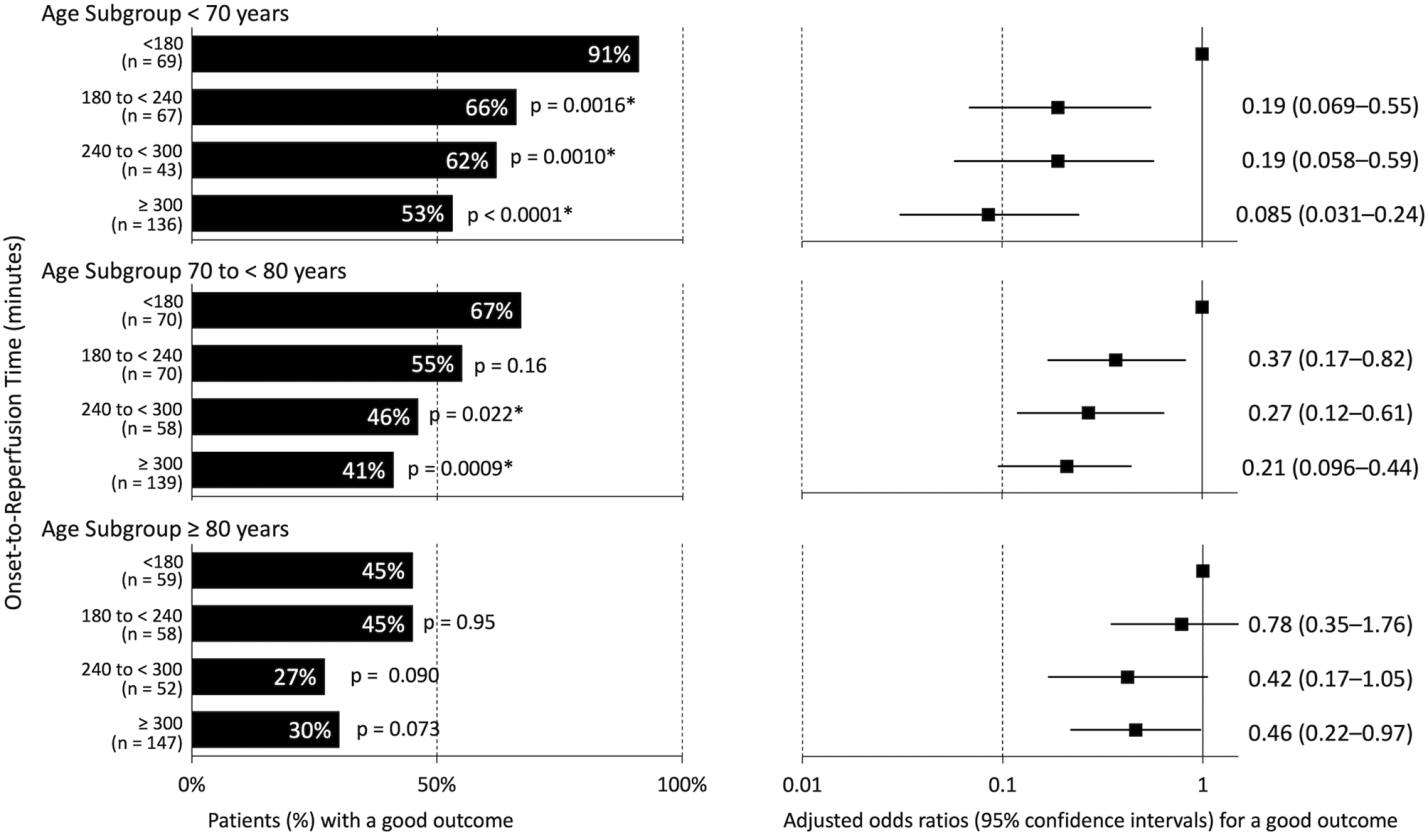
Proportions of a good outcome and adjusted odds ratios for a good outcome according to onset-to-reperfusion time (ORT) categories in each age subgroup. The proportion of a good outcome (defined as mRS score ≤ 2) was lower in the delayed ORT categories than in those with ORT < 180 min in each age subgroup, although the association was marginal in patients aged ≥ 80 years.

Of the 1010 patients with successful reperfusion, we analyzed 897 patients with pre-stroke mRS scores ≤ 2. In patients aged < 70 years and 70 to < 80 years, the proportion of good outcomes was higher in the shorter ORT categories (Fig. 3). The adjusted odds ratios for a good outcome per 1-category delay in ORT were 0.52 (95% CI 0.39–0.68) in patients aged < 70 years, 0.62 (95% CI 0.49–0.78) in patients aged 70 to < 80 years, and 0.76 (95% CI 0.60–0.96) in patients aged ≥ 80 years (Table 2). Using an ORT of < 180 min as a reference, the adjusted odds ratios for a good outcome in patients with an ORT of 180 to < 240, 240 to < 300, and ≥ 300 min were as follows: in patients aged ≤ 70 years, 0.19 (95% CI 0.069–0.55), 0.19 (95% CI 0.058–0.59), and 0.085 (95% CI 0.031–0.24); in patients aged 70 to < 80 years, 0.37 (95% CI 0.17–0.82), 0.27 (95% CI 0.12–0.61), and 0.21 (95% CI 0.096–0.44); and in patients aged ≥ 80 years, 0.78 (95% CI 0.35–1.76), 0.42 (95% CI 0.17–1.05), and 0.46 (95% CI 0.22–0.97), respectively (Fig. 3).

Although a marginal association between delayed ORT and mortality was observed in patients aged 70 to < 80 years, no associations between ORT and mortality were found in the other age subgroups. The adjusted odds ratios for mortality per 1-category delay in ORT were 1.36 (95% CI 0.82–2.26) in patients aged < 70 years, 1.59 (95% CI 1.01–2.50) in patients aged 70 to < 80 years, and 0.88 (95% CI 0.63–1.22) in patients aged ≥ 80 years (Table 2). There was no heterogeneity with respect to the age subgroups (interaction *P* = 0.054). Differences in the mortality rate among the ORT categories were found in patients aged < 70 years, but not in patients aged 70 to < 80 or ≥ 80 years (Supplementary Table S4).

### Safety outcomes

The adjusted odds ratios for sICH per 1-category delay in ORT were 0.98 (95% CI 0.57–1.69) in patients aged < 70 years, 1.21 (95% CI 0.72–2.03) in patients aged 70 to < 80 years, and 0.90 (95% CI 0.58–1.41) in patients aged ≥ 80 years. (Table 2). There was no heterogeneity with respect to age subgroups (interaction *P* = 0.75). No difference in the rate of sICH among the ORT categories was observed in any age subgroup (Supplementary Table S4).

### Sensitivity analysis

After the imputation of missing data using the multiple imputation method, the adjusted common odds ratios for better outcomes per 1-category delay in ORT were 0.66 (95% CI 0.55–0.79) in patients aged < 70 years, 0.67 (95% CI 0.56–0.79) in patients aged 70 to < 80 years, and 0.79 (95% CI 0.67–0.93) in patients aged ≥ 80 years (Supplementary Table S5), which also showed a significant time–outcome relationship in all age subgroups. These adjusted common odds ratios were not different among the age subgroups (interaction *P* = 0.087).

Of the 1010 patients, we analyzed 700 patients with an OPT of ≤ 6 h. Obvious time–outcome relationships were observed across all age subgroups. The adjusted common odds ratios for better outcomes per 1-category delay in ORT were 0.64 (95% CI 0.52–0.79) in patients aged < 70 years, 0.62 (95% CI 0.51–0.75) in patients aged 70 to < 80 years, and 0.74 (95% CI 0.60–0.91) in patients aged ≥ 80 years (Supplementary Table S5). These adjusted common odds ratios were not different among the age subgroups (interaction *P* = 0.24). The adjusted common odds ratios for better outcomes per 30-min delay in ORT were 0.89 (95% CI 0.82–0.97) in patients aged < 70 years, 0.85 (95% CI 0.79–0.92) in patients aged 70 to < 80 years, and 0.89 (95% CI 0.82–0.96) in patients aged ≥ 80 years (Supplementary Table S5). These adjusted common odds ratios were not different among the age subgroups (interaction *P* = 0.90).

In patients who underwent both CT and DWI before endovascular therapy in patients with anterior circulation occlusion, we used ASPECTS on CT for sensitivity analysis instead of ASPECTS on DWI. The adjusted common odds ratios for better outcomes per 1-category delay in ORT were 0.64 (95% CI 0.54–0.78) in patients aged < 70 years, 0.66 (95% CI 0.56–0.79) in patients aged 70 to < 80 years, and 0.83 (95% CI 0.70–0.99) in patients aged ≥ 80 years (Supplementary Table S5). These adjusted common odds ratios were significantly different among the age subgroups (interaction *P* = 0.015).

## Discussion

In this current post-hoc analysis of a large clinical registry of patients with acute cerebral large-vessel occlusion, earlier reperfusion was associated with better outcomes across all age subgroups. To the best of our knowledge, this is the first report to show the time–outcome relationship in elderly patients using a large clinical registry. Our findings confirmed that achieving early successful reperfusion is important in patients of all ages.

Previous studies have investigated the association between ORT and outcomes in different age subgroups; however, the results in the elderly subgroup differed between studies^13,14^. One of two studies showed that ORT was independently associated with a favorable outcome at 90 days in 34 patients aged ≥ 80 years^13^, whereas the other study did not find an association between ORT and a good functional outcome in 78 patients aged > 80 years with mTICI of 2b or 3^14^. In elderly patients, because of the severe stroke outcome^11^, it is difficult to show a small difference according to ORT. Given the retrospective nature of the study, an age-related selection bias for endovascular therapy may have influenced the difficulty in demonstrating the differences. The present study is important in that it showed the time–outcome relationship in patients of all ages, although the relationship was small and partial in elderly patients. Logistic regression models for primary outcome (mRS at 90 days) and good outcome (mRS ≤ 2 at 90 days) showed heterogeneity among age subgroups; however, the time–outcome relationship existed across all age subgroups (Table 2). In patients aged ≥ 80 years, the proportion of good outcomes according to ORT categories was not significantly different, but was significantly smaller in patients with ORT ≥ 300 min after adjustment for confounders (Fig. 3). In patients with OPT of 6 h or less, shorter time to reperfusion was consistently associated with better outcomes across all age subgroups without heterogeneity (Supplementary Table S5). Thus, a shorter time to reperfusion is important for all ages.

We assessed the effects of early reperfusion in consecutive patients who were hospitalized within 24 h of the onset of acute large-vessel occlusion. However, randomized clinical trials using imaging-based selection criteria showed that there was no time–outcome relationship in patients with late hospital arrival^23,24^. Furthermore, a large database showed that the time–outcome relationships were stronger in patients with an OPT of ≤ 270 min than in those with an OPT of > 270 min^10^. Based on these previous reports, we set ORT categories with thresholds of 180, 240, and 300 min, and performed a sensitivity analysis in patients with an OPT of ≤ 6 h. Consequently, we focused on the early time window and observed consistent time–outcome relationships across all ages. However, we did not intend to determine the time limit for endovascular therapy. Our registry previously revealed that endovascular reperfusion therapy is effective, regardless of the onset-to-door time^15^.

Although a marginal association of early reperfusion with survival was observed in patients aged 70 to < 80 years, no associations were noted between ORT and mortality in the other age subgroups. In addition, no association between ORT and sICH was found in any age subgroup. Previous reports from the meta-analysis of the HERMES collaborators and a large practical database documented that both mortality and sICH were not associated with the onset-to-randomization time or OPT^1,12^, which is consistent with our current results. However, in patients aged ≥ 80 years, the odds ratios for mortality and sICH tended to be low. This may also be due to an age-related selection bias for endovascular therapy.

The clinical background characteristics in the present study were not different from those in previous reports. Male sex^13,14^, hypertension^13^, cardiogenic embolism^14^, and high NIHSS score^14^ have been reported to be associated with elderly patients undergoing endovascular thrombectomy. Early hospital arrival was reported to be associated with atrial fibrillation, a high NIHSS score, and ambulance use in ischemic stroke patients^28^. This may be because atrial fibrillation is associated with stroke severity^29^. In our cohort, the proportion of patients with ASPECTS ≥ 6 in patients aged < 70 years and the proportion of those with ICA or M1 segment occlusion in patients aged of 70 to < 80 years was different among the ORT categories, although there were no trends according to ORT.

Several limitations of the current study should be noted. First, this was an observational study; thus, the selection of endovascular therapy depended on the practicing physician. Moreover, patients with unsuccessful reperfusion were excluded from the analysis. Furthermore, 8% of patients were excluded from the analysis owing to missing data. These factors may have led to a potential selection bias, although we conducted sensitivity analyses. Second, although we systematically registered stroke patients with acute large-vessel occlusion over a 2-year period at 46 centers, the sample size was not large enough to fully evaluate the time–outcome effect in each age subgroup. Third, the imaging methods were not standardized and perfusion imaging was not analyzed in this study. We used two types of ASPECTS: ASPECTS on CT, an ASPECTS on DWI in patients with anterior circulation occlusion. Although we conducted a sensitivity analysis, this was not sufficient to adjust for the impact of imaging on the outcome. Therefore, a study with modern standardized imaging methods is needed to evaluate the impact of time to reperfusion. Fourth, the assessments of the mRS scores could be biased, as acute treatment was not completely masked although the assessments were performed by independent physicians. Fifth, the assessments of the mTICI scores could be biased. A previous report revealed a discrepancy in the assessment of the mTICI score between local operators and independent core laboratories^30^. This may have led to a selection bias.

## Conclusions

Our large prospective registry of acute large-vessel occlusion revealed that early reperfusion was associated with better outcomes across all age subgroups. Achieving early successful reperfusion is important in patients of all ages, even in octogenarians.

## Supplementary information

Supplementary Information.

## Data Availability

The data, analytic methods, and study materials will not be made available to other researchers for the purpose of reproducing the results or replicating the procedure.
