# Author Correction: Time–outcome relationship in acute large-vessel occlusion exists across all ages: subanalysis of RESCUE-Japan Registry 2

**DOI:** 10.1038/s41598-021-97242-2

**Published:** 2021-09-08

**Authors:** Kenichi Todo, Shinichi Yoshimura, Kazutaka Uchida, Hiroshi Yamagami, Nobuyuki Sakai, Haruhiko Kishima, Hideki Mochizuki, Masayuki Ezura, Yasushi Okada, Kazuo Kitagawa, Kazumi Kimura, Makoto Sasaki, Norio Tanahashi, Kazunori Toyoda, Eisuke Furui, Yuji Matsumaru, Kazuo Minematsu, Takaya Kitano, Shuhei Okazaki, Tsutomu Sasaki, Manabu Sakaguchi, Masatoshi Takagaki, Takeo Nishida, Hajime Nakamura, Takeshi Morimoto, RESCUE-Japan Registry 2 Investigators

**Affiliations:** 1 Stroke Center, Osaka University Hospital, Suita, Osaka Japan; 2 Department of Neurosurgery, Hyogo College of Medicine, Nishinomiya, Hyogo Japan; 3 Department of Stroke Neurology, National Hospital Organization Osaka National Hospital, Osaka, Japan; 4 Department of Cerebrovascular Medicine, National Cerebral and Cardiovascular Center, Suita, Osaka Japan; 5 Department of Neurosurgery, Kobe City Medical Center General Hospital, Kobe, Hyogo Japan; 6 Department of Neurosurgery, National Hospital Organization Sendai Medical Center, Sendai, Miyagi Japan; 7 Cerebrovascular Center, National Hospital Organization Kyushu Medical Center, Fukuoka, Japan; 8 Department of Neurology, Tokyo Women’s Medical University, Tokyo, Japan; 9 Department of Neurological Science, Graduate School of Medicine, Nippon Medical School, Tokyo, Japan; 10 Institute for Biomedical Sciences, Iwate Medical University, Morioka, Iwate Japan; 11 Department of Neurology, Saitama Medical University International Medical Center, Hidaka, Saitama Japan; 12 Department of Stroke Neurology, Saiseikai Toyama Hospital, Toyama, Japan; 13 Division for Stroke Prevention and Treatment, Department of Neurosurgery, University of Tsukuba, Tsukuba, Ibaraki Japan; 14 Department of Clinical Epidemiology, Hyogo College of Medicine, Nishinomiya, Hyogo Japan

Correction to: *Scientific Reports* 10.1038/s41598-021-92100-7, published online 17 June 2021

The original version of this Article contained errors in Figure 3, where the x-axis label for the graph showing “Patients (%) with a good outcome” was obstructed by an image element.

The original Figure 3 and accompanying legend appear below.

**Figure 3:**
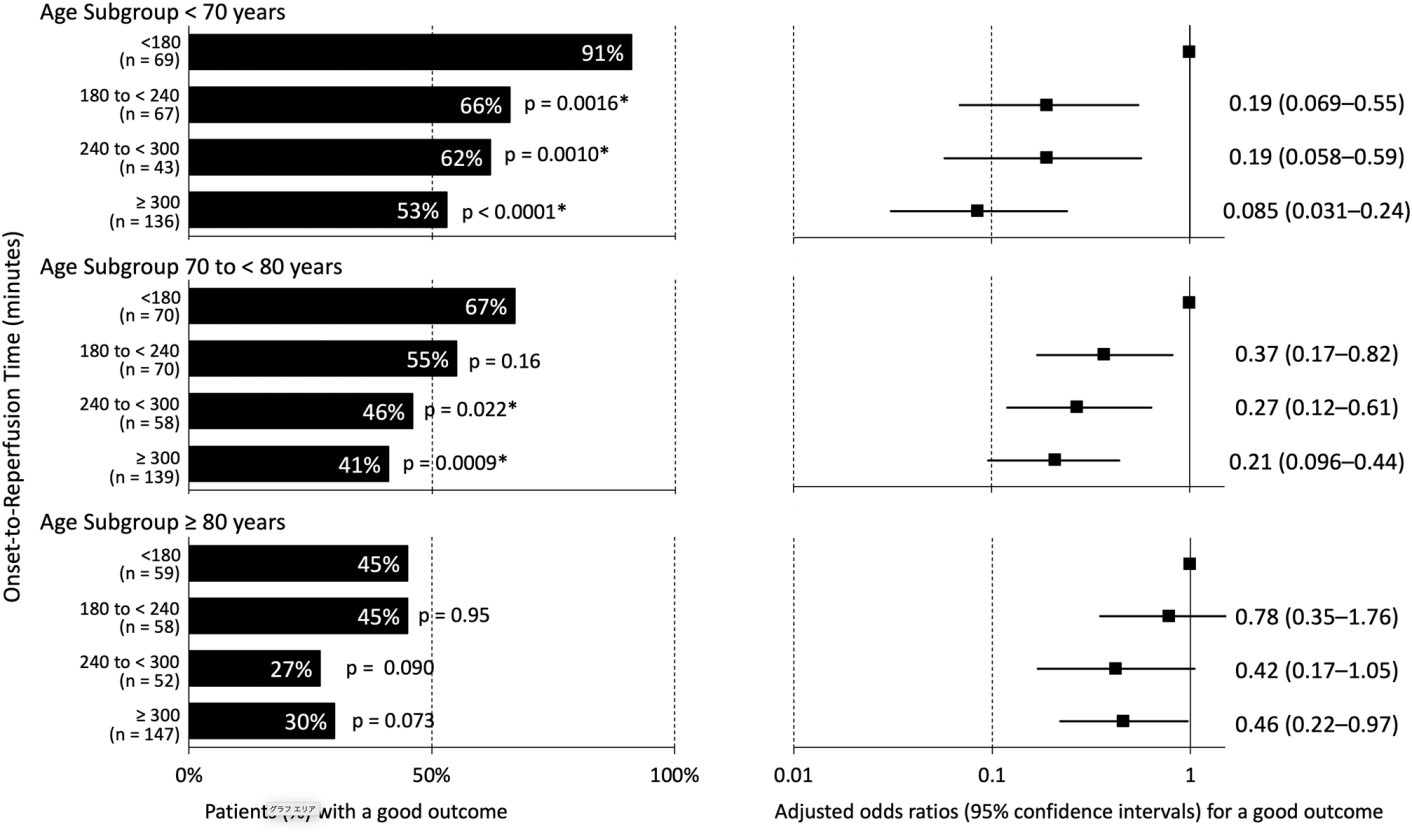
Proportions of a good outcome and adjusted odds ratios for a good outcome according to onset-to-reperfusion time (ORT) categories in each age subgroup. The proportion of a good outcome (defined as mRS score ≤ 2) was lower in the delayed ORT categories than in those with ORT < 180 min in each age subgroup, although the association was marginal in patients aged ≥ 80 years.

The original Article has been corrected.

