# Supplementary material for: Time–outcome relationship in acute large-vessel occlusion exists across all ages: subanalysis of RESCUE-Japan Registry 2
Source: Sci Rep. 2021 Jun 17;11:12782. doi: 10.1038/s41598-021-92100-7 (PMC8211644; doi:10.1038/s41598-021-92100-7)
Supplement: Supplementary file 1 — Supplementary Information. [file 41598_2021_92100_MOESM1_ESM.pdf]

Supplementary Information

Time-Outcome Relationship in Acute Large-Vessel Occlusion Exists across All Ages
# – Subanalysis of RESCUE-Japan Registry 2 –

Kenichi Todo, Shinichi Yoshimura, Kazutaka Uchida, Hiroshi Yamagami,
Nobuyuki Sakai, Haruhiko Kishima, Hideki Mochizuki, Masayuki Ezura,
Yasushi Okada, Kazuo Kitagawa, Kazumi Kimura, Makoto Sasaki, Norio Tanahashi,
Kazunori Toyoda, Eisuke Furui, Yuji Matsumaru, Kazuo Minematsu, Takaya Kitano,
Shuhei Okazaki1, Tsutomu Sasaki, Manabu Sakaguchi, Masatoshi Takagaki,
Takeo Nishida, Hajime Nakamura, Takeshi Morimoto

Supplementary Methods

Multiple Imputation Method

We developed multivariate logistic regression models using the multiple imputation method using the 1094 patients data with successful reperfusion. We imputed missing data with multiple imputation method. The multiple imputation method was performed with the aregImpute function in rms package of R software. We replaced each missing value with a set of substituted plausible values by creating 5 filled-in complete data sets using flexible additive imputation models with predictive mean matching [1]. In the imputation process, the following covariates were used to create 5 complete data sets: ASPECTS, NIHSS score, pre-stroke mRS score, IV-rtPA use, site of the main occlusions (anterior or posterior circulation) and vessels of the main occlusions (internal carotid artery and M1 segment of the middle cerebral artery occlusion or other arteries). We developed multivariate logistic regression models using the multiple imputation methods by adjusting for the clinically relevant factors as follows: ASPECTS, NIHSS score, pre-stroke mRS score, use of IV-rtPA, site of the main occlusions (anterior or posterior circulation) and vessels of the main occlusions (internal carotid artery and M1 segment of the middle cerebral artery occlusion or other arteries).

Reference:

[1] Harrell FE: Regression Modeling Strategies with Applications to Linear Models, Logistic Regression, and Survival Analysis. New York: Springer-Verlag 2001.

List of Participating Centers and Investigators

Kazunori Toyoda—Department of Cerebrovascular Medicine, National Cerebral and Cardiovascular Center; Masataka Takeuchi—Department of Neurosurgery, Seisho Hospital; Masafumi Morimoto—Department of Neurosurgery, Yokohama Shintoshi Neurosurgical Hospital; Toshiyuki Onda—Department of Neurosurgery, Sapporo Shiroishi Memorial Hospital; Masunari Shibata—Department of Neuroendovascular Treatment, Japanese Red Cross Ise Hospital; Shinichi Yoshimura–Department of Neurosurgery, Hyogo College of Medicine; Nobuyuki Sakai—Department of Neurosurgery, Kobe City Medical Center General Hospital; Takahiro Ohta—Department of Neurosurgery, Tokyo Metropolitan Tama Medical Center; Keisuke Imai—Department of Neuroendovascular Treatment, Japanese Red Cross Kyoto Daiichi Hospital; Ryo Itabashi—Department of Stroke Neurology, Kohnan Hospital; Masayuki Ezura—Department of Neurosurgery, National Hospital Organization Sendai Medical Center; Taro Yamashita—Department of Neurosurgery, Shimizu Hospital; Norihito Fukawa—Department of Neurosurgery, Kinki University; Naoto Kimura—Department of Neurosurgery, Iwate Prefectural Central Hospital; Ryosuke Doijiri—Department of Neurology, Iwate Prefectural Central Hospital; Hajime Ohta—Department of Neurosurgery, Miyakonojo Medical Association Hospital; Yukiko Enomoto—Department of Neurosurgery, Gifu University Hospital; Chisaku Kanbayashi—Department of Neurosurgery, Kawasaki Saiwai Hospital; Ikuya Yamaura—Department of Neurosurgery, Yoshida Hospital and Cerebrovascular Research Institute; Hideyuki Ishihara—Department of Neurosurgery, Yamaguchi University Hospital; Yuki Kamiya—Department of Neurology, Showa University Koto-Toyosu Hospital; Makoto Hayase—Department of Neurosurgery, Fukui Red Cross Hospital; Kouhei Nii—Department of Neurosurgery, Fukuoka University Chikushi Hospital; Junya Kobayashi—Department of Vascular Neurology, Osaka-Minami Medical Center; Hiroaki Yasuda—Department of Neurosurgery, Yamaguchi Prefectural Grand Medical Center; Ryushi Kondo, Daisuke Yamamoto—Department of Neurosurgery, Kitasato University Hospital; Manabu Sakaguchi—Department of Neurology, Osaka University Hospital; Junichiro Satomi—Department of Neurosurgery, Tokushima University Hospital; Yoshiki Yagita—

Department of Stroke Medicine, Kawasaki Medical School Hospital; Akira Handa—Department of Neurosurgery, Kurashiki Central Hospital; Atsushi Shindo—Department of Neurological Surgery, Kagawa University Faculty of Medicine; Nagayasu Hiyama—Department of Neurosurgery, Goshi Hospital; Naoki Toma—Department of Neurosurgery, Mie University Hospital; Tomoyuki Tsumoto—Cerebrovascular Center, National Hospital Organization Kyushu Medical Center; Kazumi Kimura—Department of Neurological Science, Graduate School of Medicine, Nippon Medical School; Wataro Tsuruta—Department of Neurology, Toranomon Hospital; Keigo Matsumoto—Department of Neurosurgery, Kobe Central Hospital; Yoshihiro Kiura—Department of Neurology, Hiroshima Prefectural Hospital; Takaaki Yamazaki—Department of Neurosurgery, Hakodate Neurosurgical Hospital; Taketo Hatano—Department of Neurosurgery, Kokura Kinen Hospital; Yoshihisa Matsumoto—Department of Neurosurgery, Tanushimaru Central Hospital; Takao Kojima—Department of Neurosurgery, Japanese Red Cross Nagoya Daini Hospital; Norio Ikeda—Department of Neurosurgery, Ube-kohsan Central Hospital; Shigeyuki Sakamoto—Department of Neurosurgery, Hiroshima University Hospital; Hiroyuki Ohnishi—Department of Neurosurgery, Osaka Medical College Hospital; Koichi Haraguchi—Department of Neurosurgery, Hakodate Shintoshi Hospital; Naoyuki Uchiyama—Department of Neurosurgery, Kanazawa University.

Supplementary Tables

Supplementary Table S1: Clinical characteristics according to onset-to-reperfusion time in ages < 70 years (N = 303)

	**Onset-to-Reperfusion Time (minutes)**				
	**< 180 (n = 66)**	**≥ 180 to < 240 (N = 67)**	**≥ 240 to < 300 (N = 41)**	**≥ 300 (N = 129)**	***P* Value**
Age, median (IQR), y	64 (57–67)	63 (57–67)	61 (56–67)	64 (57–67)	0.88
Male sex, No. (%)	46 (70%)	50 (75%)	28 (68%)	102 (79%)	0.38
Hypertension, No. (%)	28 (42%)	33 (49%)	17 (41%)	62 (48%)	0.76
Diabetes mellitus, No. (%)	11 (17%)	17 (25%)	8 (20%)	27 (21%)	0.66
Atrial fibrillation, No. (%)	30 (45%)	31 (46%)	21 (51%)	37 (29%)	0.012
Pre-stroke mRS score ≥ 2, No. (%)	64 (97%)	65 (97%)	39 (95%)	123 (95%)	0.90
NIHSS score, median (IQR) (N = 324)	18 (14–23)	18 (12–22)	16 (11–25)	14 (10–19)	0.015
Blood glucose, median (IQR), mg/mL (N = 296)	124 (109–150)	123 (112–156)	129 (108–177)	128 (110–156)	0.94
Systolic blood pressure, median (IQR), mmHg (N = 297)	150 (136–162)	150 (127–173)	151 (123–165)	150 (129–168)	0.87
Location of occlusion					
Anterior circulation, No. (%)	62 (94%)	61 (91%)	33 (80%)	115 (89%)	0.17
ICA or M1 segment MCA, No. (%)	45 (68%)	51 (76%)	22 (54%)	92 (71%)	0.091
IV-rtPA therapy, No. (%)	50 (76%)	46 (69%)	24 (59%)	38 (29%)	< 0.0001
ASPECTS ≥ 6, No. (%)	61 (92%)	55 (82%)	28 (68%)	106 (82%)	0.017

ASPECTS, Alberta Stroke Program Early Computed Tomography Score; BA, basilar artery; ICA, internal carotid artery; IQR, interquartile rage; IV-rtPA, intravenous recombinant tissue plasminogen activator; MCA, middle cerebral artery; mRS, modified Rankin Scale; NIHSS, National Institutes of Health Stroke Scale

Supplementary Table S2: Clinical characteristics according to onset-to-reperfusion time in ages 70 to <80 years (N = 337)

	Onset-to-Reperfusion Time (minutes)				
	< 180 (n = 71)	≥ 180 to < 240 (N = 69)	≥ 240 to < 300 (N = 58)	≥ 300 (N = 139)	*P* Value
Age, median (IQR), y	75 (73–77)	74 (73–77)	74 (72–77)	75 (73–78)	0.26
Male sex, No. (%)	48 (68%)	41 (59%)	42 (72%)	88 (63%)	0.44
Hypertension, No. (%)	46 (65%)	36 (52%)	33 (57%)	89 (64%)	0.31
Diabetes mellitus, No. (%)	14 (20%)	10 (14%)	13 (22%)	34 (22%)	0.41
Atrial fibrillation, No. (%)	49 (69%)	35 (51%)	28 (48%)	70 (50%)	0.041
Pre-stroke mRS score ≥ 2, No. (%)	64 (90%)	62 (90%)	54 (93%)	130 (94%)	0.73
NIHSS score, median (IQR)	20 (16–25)	17 (13–22)	18 (13–22)	17 (11–22)	0.018
Blood glucose, median (IQR), mg/mL (N = 327)	130 (111–152)	121 (104–159)	140 (116–184)	128 (108–150)	0.15
Systolic blood pressure, median (IQR), mmHg (N = 326)	159 (136–175)	147 (134–157)	149 (135–164)	159 (138–173)	0.032
Location of occlusion					
Anterior circulation, No. (%)	68 (96%)	63 (91%)	54 (93%)	125 (90%)	0.51
ICA or M1 segment MCA, No. (%)	57 (80%)	46 (67%)	51 (88%)	106 (76%)	0.035
IV-rtPA therapy, No. (%)	44 (62%)	43 (62%)	44 (76%)	32 (23%)	< 0.0001
ASPECTS ≥ 6, No. (%)	57 (80%)	59 (86%)	48 (83%)	112 (81%)	0.82

ASPECTS, Alberta Stroke Program Early Computed Tomography Score; BA, basilar artery; ICA, internal carotid artery; IQR, interquartile rage; IV-rtPA, intravenous recombinant tissue plasminogen activator; MCA, middle cerebral artery; mRS, modified Rankin Scale; NIHSS, National Institutes of Health Stroke Scale

Supplementary Table S3: Clinical characteristics according to onset-to-reperfusion time in ages ≥ 80 years (N = 370)

	Onset-to-Reperfusion Time (minutes)				
	**< 180 (n = 67)**	**≥ 180 to < 240 (N = 72)**	**≥ 240 to < 300 (N = 60)**	**≥ 300 (N = 171)**	***P* Value**
Age, median (IQR), y	84 (82–87)	84 (82–88)	85 (82–87)	85 (82–87)	0.96
Male sex, No. (%)	23 (34%)	34 (47%)	23 (38%)	78 (46%)	0.31
Hypertension, No. (%)	40 (60%)	49 (68%)	36 (60%)	115 (67%)	0.54
Diabetes mellitus, No. (%)	9 (13%)	13 (18%)	10 (17%)	27 (16%)	0.90
Atrial fibrillation, No. (%)	45 (67%)	48 (67%)	37 (62%)	102 (60%)	0.62
Pre-stroke mRS score ≥ 2, No. (%)	53 (79%)	56 (78%)	45 (75%)	142 (82%)	0.54
NIHSS score, median (IQR)	19 (15–23)	18 (14–22)	19 (15–26)	18 (14–23)	0.51
Blood glucose, median (IQR), mg/mL (N = 361)	124 (108–149)	132 (110–156)	128 (105–154)	127 (112–157)	0.46
Systolic blood pressure, median (IQR), mmHg (N = 358)	156 (137–172)	162 (140–176)	158 (135–170)	160 (142–172)	0.78
Location of occlusion					
Anterior circulation, No. (%)	64 (96%)	66 (92%)	60 (100%)	157 (92%)	0.11
ICA or M1 segment MCA, No. (%)	49 (73%)	52 (72%)	49 (82%)	131 (77%)	0.58
IV-rtPA therapy, No. (%)	43 (64%)	50 (69%)	35 (58%)	33 (19%)	< 0.0001
ASPECTS ≥ 6, No. (%)	58 (87%)	64 (89%)	50 (83%)	145 (85%)	0.80

ASPECTS, Alberta Stroke Program Early Computed Tomography Score; BA, basilar artery; ICA, internal carotid artery; IQR, interquartile rage; IV-rtPA, intravenous recombinant tissue plasminogen activator; MCA, middle cerebral artery; mRS, modified Rankin Scale; NIHSS, National Institutes of Health Stroke Scale

Supplementary Table S4: The proportions of mortality and symptomatic intracranial hemorrhage according to onset-to-reperfusion time

	Onset-to-Reperfusion Time (minutes)				
**Age Subgroups**	**< 180**	**≥ 180 to < 240**	**≥ 240 to < 300**	**≥ 300**	***P* Value**
**Mortality at 90 days (N = 1010)**					
**< 70 years (N = 303)**	0% (0/66)	7% (5/67)	15% (6/41)	5% (7/129)	0.018
**70 to < 80 years (N = 337)**	1% (1/71)	7% (5/69)	5% (3/58)	9% (13/139)	0.17
**≥ 80 years (N = 370)**	12% (8/67)	7% (5/72)	13% (8/60)	8% (13/171)	0.43
**Symptomatic intracranial hemorrhage within 72 hours (N = 951)**					
**< 70 years (N = 282)**	2% (1/63)	8% (5/63)	8% (3/37)	3% (4/119)	0.23
**70 to < 80 years (N = 319)**	0% (0/67)	9% (6/68)	4% (2/55)	6% (8/129)	0.10
**≥ 80 years (N = 350)**	6% (4/64)	4% (3/68)	8% (4/52)	4% (7/166)	0.75

Supplementary Table S5: Adjusted odds ratios for outcomes according to ORT in sensitivity analysis

Outcome	Subjects	Effect Variable	Age Subgroups	Adjusted Values[1] (95% CI)	P for interaction
**mRS score at 90 days**	1094 patients with mTICI scores ≤ 2b using multiple imputation method	Common odds ratios per 1-category delay in ORT[2]	< 70 years (N = 327)	0.66 (0.55–0.79)	0.087
			70 to < 80 years (N = 370)	0.67 (0.56–0.79)	
			≥ 80 years (N = 397)	0.79 (0.67–0.93)	
**mRS score at 90 days**	700 patients with mTICI scores ≤ 2b and onset-to-puncture time ≤ 6 hours	Common odds ratios per 1-category delay in ORT[2]	< 70 years (N = 221)	0.64 (0.52–0.79)	0.24
			70 to <80 years (N = 240)	0.62 (0.51–0.75)	
			≥ 80 years (N = 239)	0.74 (0.60–0.91)	
**mRS score at 90 days**	700 patients with mTICI scores ≤ 2b and onset-to-puncture time ≤ 6 hours	Common odds ratios per 30-minute delay in ORT[3]	< 70 years (N = 221)	0.89 (0.82–0.97)	0.90
			70 to <80 years (N = 240)	0.85 (0.79–0.92)	
			≥ 80 years (N = 239)	0.89 (0.82–0.96)	
**mRS score at 90 days**	1010 patients with mTICI scores ≤ 2b	Common odds ratios per 1-category delay in ORT[2]	< 70 years (N = 327)	0.64 (0.54–0.78)[4]	0.015
			70 to < 80 years (N = 370)	0.66 (0.56–0.79)[4]	
			≥ 80 years (N = 397)	0.83 (0.70–0.99)[4]	

CI, confidence interval; mRS, modified Rankin Scale; mTICI, modified thrombolysis in cerebral infarction; ORT, onset-to-reperfusion time.

[1]Adjustment was made for the Alberta Stroke Program Early Computed Tomography Score (ASPECTS) (≥6 or < 6), National Institutes of Health Stroke Scale score, pre-stroke modified Rankin Scale score, use of intravenous recombinant tissue plasminogen activator, the site of the main occlusions (anterior or posterior circulation), and vessels of the main occlusions (internal carotid artery and M1 segment of the middle cerebral artery occlusion or other arteries). ASPECTS was derived from computed tomography (CT) or magnetic resonance diffusion-weighted imaging (DWI). If both CT and DWI were performed before endovascular therapy, ASPECTS on DWI was used for the analysis. In patients with stroke in the posterior circulation, the posterior circulation ASPECTS on DWI was used for the analysis.

[2]ORT was divided into 4 categories: < 180, 180 to < 240, 240 to < 300, and ≥ 300 minutes.

[3]ORT was entered as a continuous variable.

[4]If both CT and DWI were performed before endovascular therapy, ASPECTS on CT was used for the analysis.
